# A technology platform for standardized cryoprotection and freezing of large-volume brain tissues for high-resolution histology

**DOI:** 10.3389/fnana.2023.1292655

**Published:** 2023-11-02

**Authors:** Ramdayalan Kumarasami, Richa Verma, Karthika Pandurangan, Jivitha Jyothi Ramesh, Sathish Pandidurai, Stephen Savoia, Jaikishan Jayakumar, Mihail Bota, Partha Mitra, Jayaraj Joseph, Mohanasankar Sivaprakasam

**Affiliations:** ^1^Department of Electrical Engineering, Indian Institute of Technology Madras, Chennai, India; ^2^Healthcare Technology Innovation Centre, Indian Institute of Technology Madras, Chennai, India; ^3^Sudha Gopalakrishnan Brain Centre, Indian Institute of Technology Madras, Chennai, India; ^4^Cold Spring Harbor Laboratory, Cold Spring Harbor, New York, NY, United States; ^5^Center for Computational Brain Research, Indian Institute of Technology Madras, Chennai, India

**Keywords:** freezing platform, human neuroanatomy, sucrose cryoprotection, whole brain, freezing rate, brain master pattern, high-resolution histology, cryopreparation

## Abstract

Understanding and mapping the human connectome is a long-standing endeavor of neuroscience, yet the significant challenges associated with the large size of the human brain during cryosectioning remain unsolved. While smaller brains, such as rodents and marmosets, have been the focus of previous connectomics projects, the processing of the larger human brain requires significant technological advancements. This study addresses the problem of freezing large brains in aligned neuroanatomical coordinates with minimal tissue damage, facilitating large-scale distortion-free cryosectioning. We report the most effective and stable freezing technique utilizing an appropriate choice of cryoprotection and leveraging engineering tools such as brain master patterns, custom-designed molds, and a continuous temperature monitoring system. This standardized approach to freezing enables high-quality, distortion-free histology, allowing researchers worldwide to explore the complexities of the human brain at a cellular level. Our approach combines neuroscience and engineering technologies to address this long-standing challenge with limited resources, enhancing accessibility of large-scale scientific endeavors beyond developed countries, promoting diverse approaches, and fostering collaborations.

## 1. Introduction

Understanding the whole human brain at a cellular level, particularly its connectivity, has been a long-standing question in neuroscience (Sporns et al., 2005; Sporns, 2013; Lisman, 2015). While classical approaches of anatomically studying the brain have been employed since the early 1900s (Gal and Cagle, 2005; Brodmann and Garey, 2006), the field has seen significant advancements in more recent years, particularly the development of diverse high-throughput, integrated digital histology and computational pipelines, which have been applied in rodents such as mice, rats, and primates such as marmosets (Oh et al., 2014; Pinskiy et al., 2015; Mikula, 2016; Johnson et al., 2019; Lin et al., 2019). However, a number of unique challenges arise at each increasing size scale of brains from the mouse to the human brain, and the scaling of these approaches requires standardized resource methods for high-quality and high-throughput cellular scale histology pipelines that can be implemented in labs worldwide, even with resource-limitations.

An essential step in achieving cellular-level quantification in human brains is through high-throughput histology pipelines involving the cryopreparation or freezing of large-volume brain tissues before cryosectioning. The traditional cryosectioning involves obtaining thin slices of frozen tissue (~10–50 microns) using a microtome or a cryostat (–18°C to –25°C), and subsequently staining using different dyes and examining them under a microscope (Gal and Cagle, 2005; Fischer et al., 2008). This process, however, has not been optimized for larger tissues. As the brain size scales from rodents to humans (1 cm^3^ to 1,500 cm^3^), several challenges, such as non-uniform freezing, tissue cracking, formation of freezing artifacts, and misalignment during the freezing process, become more pronounced. This problem is further exasperated in the heterogeneously diverse human brain, which has poor thermal properties with a measured thermal conductivity of 0.51 W/mK (Hasgall et al., 2022). These poor thermal properties, combined with the large size of the tissue, pose significant difficulties during the cryopreparation step of the process. Various techniques using dry ice, isopentane (2-methyl butane), and liquid nitrogen have been effective in achieving uniform freezing of smaller brains, such as those of rodents and smaller primates, but have not been evaluated or standardized for the larger brain tissues (Currle and Monuki, 2007; Pinskiy et al., 2013, 2015). The larger the tissue, the more time it takes to freeze, which increases the possibility of freezing artifacts due to ice crystal formation and results in tissue damage. One way of avoiding these artifacts is by using compounds that resist changes in volume during freezing. These compounds, called cryoprotectants, are often used to minimize these artifacts during histology. However, the choice and duration of cryoprotectant depend on the specimen size and the expected histological quality, which requires systematic evaluation for application to large brain tissues (Rosene et al., 1986; Estrada et al., 2017). Furthermore, if the histological processes are expected to be applied to the whole large volume tissues, proper brain alignment in neuroanatomical coordinates during freezing becomes vital for avoiding alignment issues during cryosectioning and specifically for studies that require histological three-dimensional (3D) reconstruction (Pinskiy et al., 2013).

Understanding brain connectivity, particularly the large-volume human brain connectivity, is a complex challenge that requires interdisciplinary and collaborative approaches across the globe. This also means that the funding required is often limited to large-scale projects, limiting it to a few developed countries. There is a significant need for affordable yet reproducible technologies that can be implemented across multiple disciplines, such as neuroscience and engineering. In this paper, we present a standardized resource method that addresses the problem of freezing large volumes of brain tissues with minimal tissue damage, thereby enabling high-throughput histology and 3D digital reconstruction. The technologies and components described in this paper are affordable, ensuring they are not limited to large-scale funded projects and are easily reproducible. The method utilizes technologies widely used in engineering and includes customized equipment, custom-designed brain-specific components, and a temperature monitoring system to enable the standardization of the freezing of large-volume brains. To evaluate our cryopreparation method, particularly the effectiveness of the various cryoprotectants, the cryogenic media, and the rates of temperature decrease required for optimal freezing, we used high-resolution digital histology on large human and animal brains as a measure of the output of the techniques.

## 2. Materials and equipment

**Figure 1:**
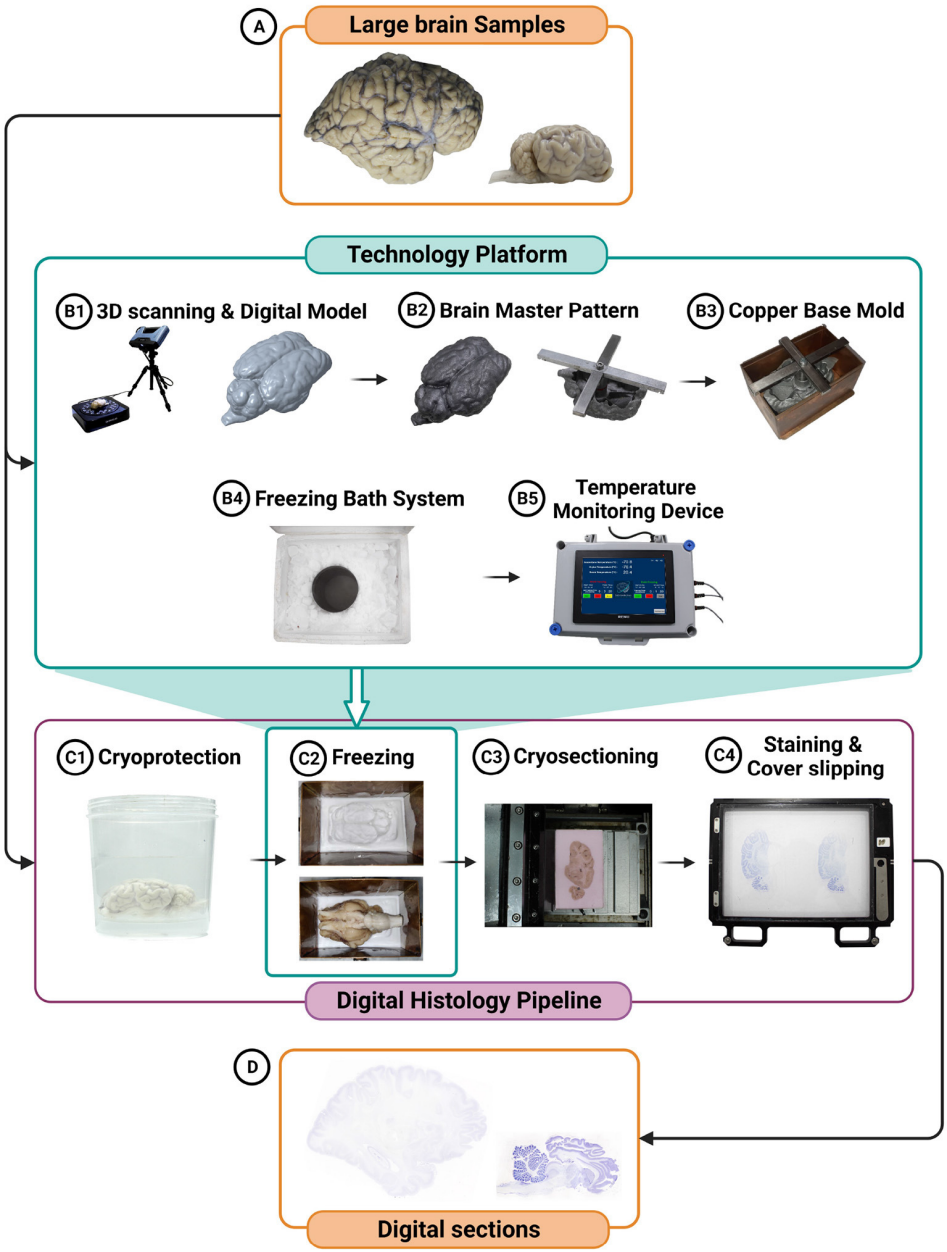
Schematic representation of the technology integration in the freezing process is depicted, featuring the creation of a digital model of the brain tissue **(A)** prior to cryoprotection **(B1)**, the generation of a sample-specific brain master pattern **(B2)**, a sample-specific copper base mold **(B3)**, the utilization of a freezing bath system **(B4)**, and the incorporation of a temperature monitoring device **(B5)**. This technology platform enables efficient freezing and monitoring while minimizing damage to the large-volume brain tissue. Additionally, the digital histology pipeline for large brain specimens, highlighting the key steps involved, including cryoprotection **(C1)**, freezing **(C2)**, cryosectioning **(C3)**, staining **(C4)**, coverslipping **(C4)**, and digitization using a scanner **(D)** is depicted.

The cryopreparation or freezing process is an integral part of a comprehensive digital histology pipeline deployed to process large brain tissues in a high throughput manner. The cryosectioning process offers several advantages over alternate methods, such as wax embedding processes, notably in terms of minimizing distortion and shrinkage due to dehydration. The cryosectioning pipeline encompasses brain extraction, fixation, cryoprotection, freezing, cryosectioning, staining, coverslipping, and generation of digital slides (see Figure 1).

### 2.1. Brain sample procurement and fixation

The experiments reported in this paper were conducted using procedures approved by the Indian Institute of Technology Madras institutional ethics committee (IEC/2021-01/MS/06). The large animal goat brains used for the experiments were obtained from commercial sources. After extraction, the goat brains were immersed in 4% paraformaldehyde (PFA, pH 7.4, monitored weekly) for two weeks for fixation. The human brain specimen reported here (25 years/ Male) was procured from the Department of Pathology, Christian Medical College (CMC), Vellore, India. For the human brain, autopsy and brain extraction (James et al., 2023) were conducted after obtaining due consent from the next of kin, following the Declaration of Helsinki, under the approved Institutional Review Board (IRB) protocol of CMC, Vellore (IRB Min. No. 13262). Post extraction, the human brain was suspended in 4% PFA. The physical dimensions and weight of the specimens were measured before processing.

### 2.2. The technology platform for freezing large brains

To ensure uniform freezing of large-volume whole brain tissues with minimal artifacts, we have developed a technology platform that has the following components: sample-specific brain master patterns, copper base molds, a freezing bath system, and a temperature monitoring system (see Figure 1).

#### 2.2.1. Brain master pattern

Master pattern is an engineering concept commonly used to create molds of components in manufacturing processes such as casting. They are designed by replicating a component or object with precision. We have applied this concept to the brain tissues, and throughout this manuscript, the brain tissue replicas are referred to as the brain master patterns. The brain master pattern is a sample-specific surface model of the brain specimen obtained using in-skull structural Magnetic Resonance Imaging (MRI) and 3D surface scans of the extracted brain (see Figure 1B1). The master pattern, designed digitally and manufactured using metal 3D printing, creates a “negative” of the tissue in the embedding medium into which the actual brain sample can be placed with precise anatomical alignment obtained from the MRI and the 3D surface scans. The brain master pattern thus helps orient the brain tissue in neuroanatomical coordinates during the cryopreparation process before cryosectioning. In our pipeline, the extracted brains were scanned using a 3D surface scanner, Einscan Pro2X 2020, prior to cryoprotection. The scanner uses structured light technology to capture the brain surface at an accuracy of 40 microns and, in combination with the in-skull MRI, was used to create a digital model (see Figure 1B1). The scanner generated a stereolithography (STL) file containing point cloud data and triangular facets approximating the brain's surface. This STL file was transformed into a more structured and information-rich 3D solid model using computer-aided design (CAD) software–Solid Edge. The resulting 3D model, now detailed and editable, becomes suitable for further processing using CAD software such as PTC Creo. To ensure optimal contact between the temperature source and the base mold, the solid brain model was oriented according to its intended placement in the base mold in the software. It is recommended to align the shortest dimension of the brain tissue with either the length or breadth of the mold. The resulting model, after minor modifications, was shelled to economize on metal 3D printing costs. A positioning bar was digitally incorporated into the 3D model for added convenience and alignment, facilitating its placement and alignment within the base mold (Figure 1B2). The digital models were 3D printed in an aluminum alloy (AlSi10Mg) that can withstand the low temperatures used in the process and were created using an EOS EOSINT M 280 Direct Metal Laser Sintering machine. Brain master patterns were designed for the large whole brain samples, i.e., the whole goat brain and the adult human brain, to maintain the appropriate sectioning planes while freezing.

#### 2.2.2. Base molds

The base molds, into which the tissue sample and the embedding medium are placed, were designed as open rectangular containers made from copper with a removable base, referred to as the base plate (Figure 1B3). The choice of copper was based on its thermal properties (high thermal conductivity of 386 W/mK) and minimal volume change during freezing. The removable base plate facilitated easy removal of the cryo block (embedded tissue sample). The base molds were created in different sizes and were made specific for each brain sample using 1 mm thick copper sheets that were laser cut and welded together. The internal dimensions of the base mold were designed to be 5–10% more than the tissue dimensions.

#### 2.2.3. Freezing bath system

The freezing bath system, into which the base molds were immersed, contains a stainless-steel container filled with chilled isopentane, which was then placed in a styrofoam box and surrounded by dry ice (Figure 1B4). To facilitate the isopentane to chill to –80°C, pulverized dry ice was directly added to the isopentane, and the mixture acted as the freezing source. The isopentane container was selected such that its diameter is 10–20% more than the copper base mold's diagonal. The styrofoam box was selected with dimensions about 20–30% more than the isopentane container dimensions.

#### 2.2.4. Temperature monitoring system

During the freezing process, monitoring the temperature of the freezing source is vital. We have implemented a continuous monitoring system (Figure 1B5) to measure the temperature of the isopentane bath and the surrounding dry ice. The system consisted of Resistance Temperature Detector (RTD) sensors (platinum glass wire wound RTD SB0920 from TE Connectivity), with an accuracy of ±0.25°C. The temperature sensors were connected to a data acquisition system (National Instruments, NI DAQ 6216–USB). The temperature time series data was recorded and logged during freezing using a custom-developed LabVIEW application.

## 3. Methods

### 3.1. Brain fixation and cryoprotection

Prior to the cryopreparation of the large brain specimens, the tissue was fixed using an appropriate fixative, such as 4% PFA in 0.01 M phosphate buffer (PB). The fixation of the tissue can be achieved through either perfusion and immersion or immersion-only methods. After the fixation process, a brain master pattern was generated using a combination of the structural MRI and 3D surface scans, as described above, following which cryoprotection of the brain tissue sample using gradient sucrose (10% sucrose in 4% PFA, followed by 20 and 30% sucrose in 0.01M PB) was performed with the endpoint of each gradient step being when the specimen was completely sunk, i.e., fully equilibrated.

### 3.2. Large brain freezing process

**Figure 2:**
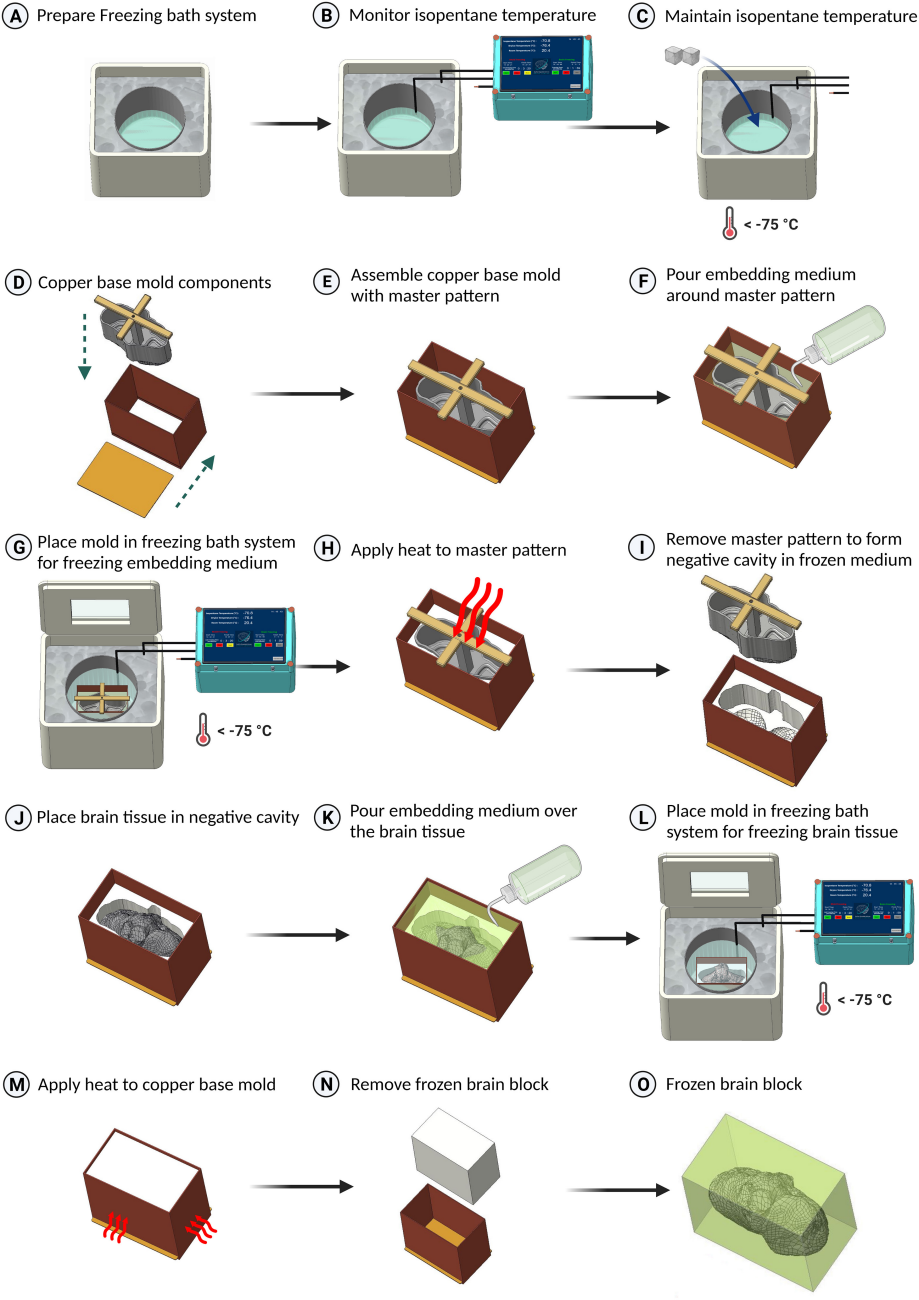
Schematic representation of the freezing protocol for large brain tissues. **(A)** Preparation of the freezing bath system, utilizing a styrofoam box filled with dry ice and a stainless steel container with isopentane. **(B)** Data logging of the isopentane temperature. **(C)** Addition of dry ice to cool the isopentane below –75°C. **(D–F)** Assembly of the brain master pattern in the copper base mold and pouring embedding medium around the master pattern. **(G)** Placement of the base mold in the isopentane container for freezing the embedding medium. **(H, I)** Application of heat to remove the master pattern from the base mold. **(J, K)** Placement of the brain tissue in the base mold and covering it with embedding medium. **(L)** Freezing the tissue by placing the copper base mold in isopentane. **(M)** Application of heat using a heat gun to the sides of the copper base mold to slightly melt the embedding medium using a heat gun. **(N)** Removal of the frozen block from the base mold. **(O)** The resulting frozen block with embedded brain tissue (embedding medium shown translucent).

The brain freezing process consisted of two important steps: the production of the cryomolds and the freezing of the brain, described below and summarized graphically in Figure 2.

#### 3.2.1. Cryomold production

The initial setup contained the dry ice-filled styrofoam box with the isopentane container (Figure 2A). A temperature sensor was fixed to the base of the isopentane container using adhesive tape. In addition, temperature sensors were placed in the dry ice as well. The isopentane was chilled to −80°C by adding dry ice. Before reaching the appropriate temperature of –80°C, isopentane boils and can potentially spill inside the base mold, causing contamination of the tissue cryoblock. To minimize this, the isopentane level within the container was pre-estimated and maintained less than the copper base mold's height. This entire process was monitored continuously with the readings from the temperature sensors to ensure that the desired temperatures were maintained (Figures 2B, C).

When the isopentane reached the appropriate temperature, the brain master pattern and the copper base mold were assembled (Figures 2D, E), along with the embedding medium (Optimal Cutting Temperature compound–Tissue Freezing Medium, Leica Biosystems India) which was poured around the master pattern up to 95% of the brain master pattern's height (Figure 2F). Special care at this step was needed to be taken to minimize the air bubbles inside the embedding medium as well as to minimize the excess embedding media falling inside the master pattern's internal cavity.

The assembled base mold with the embedding medium was placed at the center of the isopentane container, and the styrofoam box was closed from the top to prevent the influence of the warmer ambient conditions on the freezing process (Figure 2G). A transparent window with acrylic or glass on either side of the styrofoam box's cover helped visually inspect mold production. The initial placement of the base mold caused an increase in the temperature of isopentane and required the addition of dry ice to maintain the desired temperature.

Once the embedding medium within the base mold was frozen, the copper base mold was gently removed from the freezing source, and the master pattern was removed with the aid of gentle heat produced by a heat gun, which facilitated the separation from the embedding medium (Figure 2H). Once the master pattern was separated, a negative cavity with a brain surface was created in the embedding medium (Figure 2I) and helped place the brain in the required coordinates or cutting plane during freezing.

#### 3.2.2. Brain freezing

The brain tissue sample was carefully removed from the cryoprotectant and placed within the newly created negative cavity within the base mold. If placed correctly, there should not be any movement while trying to move the tissue specimen (Figure 2J). Care should be taken so that the tissue sample does not come in contact with the walls of the copper base mold, as it might cause thermal damage to the tissue. A thin coating of the embedding medium was placed on the tissue to ensure that the brain sample surface does not experience very fast and high thermal gradients. Finally, the embedding medium was poured over the brain tissue, covering all the gaps (Figure 2K).

With the isopentane maintaining its temperature at –80°C, the filled brain mold was placed inside the isopentane container (Figure 2L). Once the embedding medium was frozen and covered all areas of the brain tissue, indicated by the opaque white top surface of the frozen medium, the base mold was removed from the isopentane container. The cryoblock was removed from the base mold with the aid of gentle heat applied to the sides of the copper base mold (Figure 2M). The bottom of the base mold was removed first, and then the brain block was pushed from either the top or the bottom surface to release it from the mold (Figure 2N).

The sides of the brain cryoblock were then labeled with appropriate orientation (anterior-posterior, dorsal-ventral, right-left) (Figure 2O), and the brain block was transferred to a –80°C refrigerator and stored for a minimum of 24 h to ensure uniform freezing throughout the tissue prior to cryosectioning.

### 3.3. Cryosectioning using the tape transfer technique

A large format cryomacrotome (Leica CM 3600 XP, Leica Biosystems) was used for cryosectioning using the tape transfer technique (Pinskiy et al., 2015), which was modified and optimized for large tissue blocks. The block from –80°C storage was placed inside the cryostat for at least 30 mins to equilibrate with the chamber temperature maintained between –18°C to –20°C. The large brains were sectioned in the sagittal plane at 20-micron thickness. Every two consecutive sections were transferred to two adjacent large format 6” × 8” slides for alternate series of staining with Nissl and Hematoxylin and Eosin (H&E) (data not shown). Block Face Imaging with ultraviolet and white light was taken before each section using an excitation and emission wavelength of 254 nm (UV-C) and 400–500 nm (visible spectrum), respectively (Karthik et al., 2023).

### 3.4. Staining, coverslipping, and imaging

The slides were dried for a day and then stained for two series of histological evaluations with Nissl (Thionin 0.2%) and H&E using a custom-designed automated stainer capable of handling the large format 6” × 8” slides. Next, the slides were coverslipped with Distyrene, Plasticizer, and Xylene (DPX, Merck) mountant using a custom-developed automated cover slipper. After coverslipping, the slides were digitized at an in-plane resolution of 0.5 μm/pixel using a large format scanner (TissueScope LE120, Huron Digital Pathology, Canada). The images were converted into jpeg2000 (.jp2 format) and were transferred to a custom-designed Laboratory Inventory Management System (LIMS) where the full-resolution images underwent quality control (stitching, white balance, or focus), staining (too dark or too light), tissue quality (water droplets, damage), and coverslipping issues (presence of air and water droplets). The sections that passed through the quality control checks were included for analysis.

## 4. Results

### 4.1. Histological outcomes of the freezing protocol on the human brain

**Figure 3:**
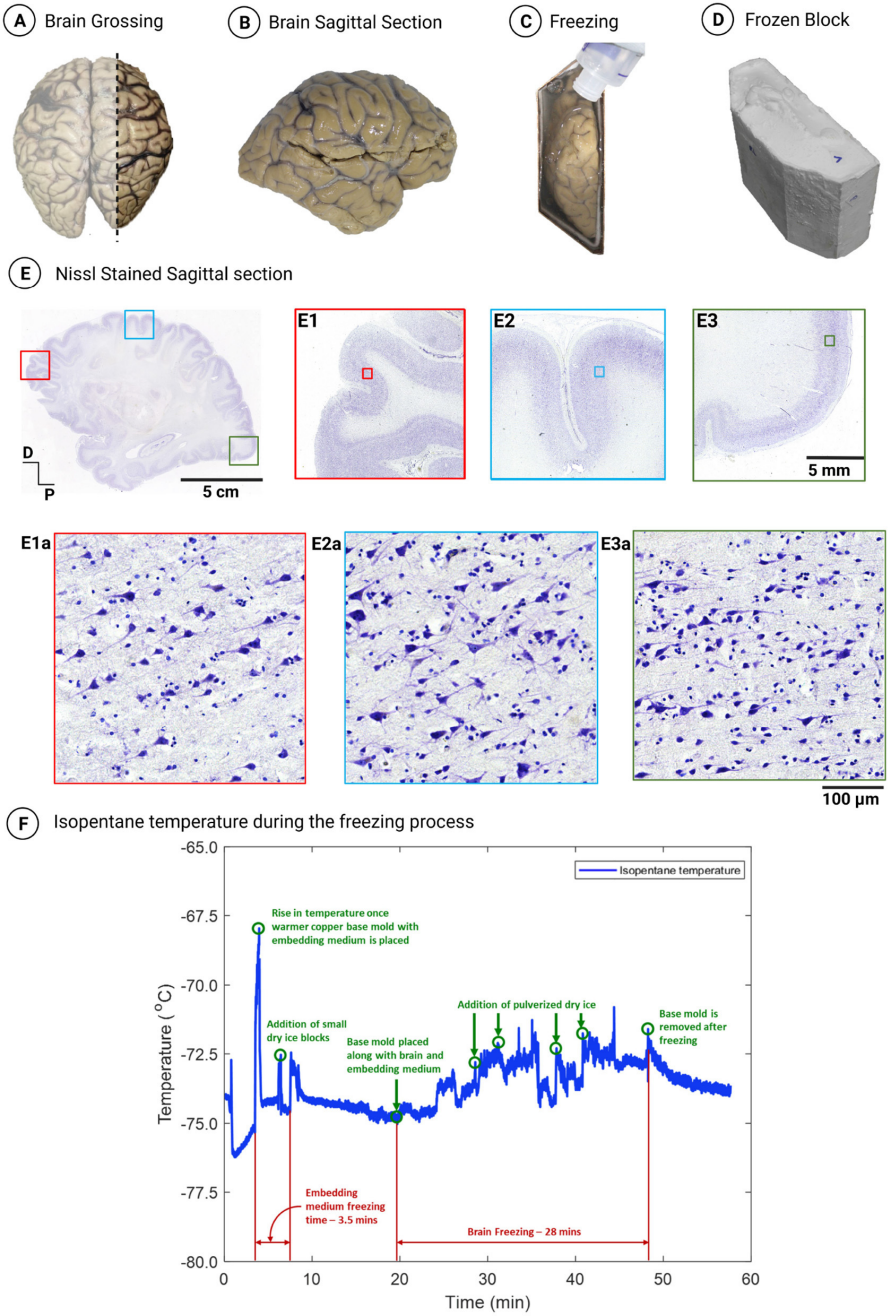
**(A)** Grossing of a sagittal section from the right hemisphere of an adult human brain. **(B)** Sagittal view of the grossed right lateral section. **(C)** Right lateral section placed in the mold during the freezing process. **(D)** Resulting frozen block of the right lateral section (796 cm^3^). **(E)** Nissl-stained sagittal section sectioned at 20 microns thickness and processed using the optimized cryoprotection protocol with gradient sucrose and frozen using an isopentane and dry ice bath with a freezing rate of 2–3°C/min. **(E1–E3)** Demonstrate the layered organization of the cortex in the frontal, dorsal, and occipital cortex, respectively, with no observed freezing artifacts. **(E1a–E3a)** Exhibit high-resolution images of the marked inset in **(E1–E3)**, respectively, showcasing multiple well-defined pyramidal neurons and granule cells with no osmotic shock. **(F)** Isopentane temperature profile during the freezing of the right lateral section.

The optimized protocol was applied to an adult brain sectioned in the sagittal plane–right hemisphere section with overall dimensions 150 × 110 × 42 mm and an estimated volume of 693 cm^3^–that was cryoprotected using gradient sucrose using the equilibrium method and frozen using the custom-designed mold in an isopentane and dry ice bath (Figures 3A–D) resulting in a cryoblock of volume 796 cm^3^. Figure 3E shows a 20-micron thick sagittal section of the right hemisphere of dimensions 15 cm anterior to posterior and 11 cm dorsal to ventral. The histological evaluation from various regions across the tissue, namely in the frontal, dorsal, and occipital cortex, demonstrated good structural integrity (Figures 3E1–3, respectively). In addition, the high-resolution images at the microstructural level from each region showed well-preserved cells with multiple well-defined neurons and other cells of the brain with no significant tissue damage (Figures 3E1a–3a). Figure 3F shows the isopentane temperature variations during the freezing of the block from which this sagittal section, outlining the excellent quality histology, was taken. As evident from the graph, the temperature was maintained at a reasonably constant level with occasional fluctuations in the isopentane temperature, which were effectively mitigated by the regular addition of dry ice, ensuring consistent and optimal cooling conditions. The tissue freezing process for this large volume block lasted 28 min, with an average isopentane temperature of –73.5°C ± 1°C.

In order to further quantify the different parameters when freezing large brain tissues without significant artifacts, we evaluated the variables on which the outcomes are highly dependent. These parameters are the choice of cryoprotectant, the duration of cryoprotection, the freezing medium or source, and the freezing rate. Therefore, systematic experiments were performed to evaluate each of the above parameters on large animal brains (goat), and the ideal choice was determined by examining the histological sections. The animal experiments allowed the monitoring of parameters both on the inside as well as the outside of the brain tissue samples.

### 4.2. Choice of cryoprotection for large brains for tape transfer based cryosectioning

Cryoprotection is achieved through diffusion by immersing the brain tissue in chemicals that replace the water in the tissue without causing expansion and maintaining the tissue's volume during freezing. We tested and compared the two most commonly used cryoprotectants—gradient sucrose or a combination of glycerol and dimethyl sulfoxide (DMSO) (Estrada et al., 2017)—for the duration of immersion and evaluated the better cryoprotectant, based on histology, for freezing large brains and cryosectioning using the tape transfer technique (Pinskiy et al., 2015). In addition, a series of experiments were done to quantify differences in the two cryoprotectants and their relative exposure time and methods utilized for freezing. The cryoprotectant immersion duration varies depending on the size of the specimen, ranging from 24 h (Pinskiy et al., 2015) to several days (Rosene et al., 1986). For instance, mouse brains are cryoprotected using a 10% sucrose solution in 4% PFA for 24 h, followed by 20% sucrose in PB for another 24 h (Pinskiy et al., 2013, 2015). Studies on monkey brains used immersion in a solution of 10% glycerol and 2% DMSO for 1–3 days, followed by immersion in a solution of 20% glycerol and 2% DMSO for 3–5 days for efficient cryoprotection (Rosene et al., 1986; Estrada et al., 2017). Variations in cryoprotection methods also exist where the specimen is directly immersed in the highest concentration, such as 30% sucrose solution in fixative or buffer (Rosene et al., 1986), or the specimen undergoes successive immersion from lower to higher concentrations of cryoprotectant. Hence, optimizing the choice of cryoprotectant and its duration based on the tissue size and histological quality is essential to minimize cellular osmotic shock and maintain the quality of histology.

### 4.3. Cryoprotection: sucrose vs. glycerol and DMSO

Post fixation with 4% PFA, we separated two goat brains along the mid-line into two hemispheres. Each hemisphere of the first goat brain (GB1) was cryoprotected, using the equilibrium protocol, where the sample was transferred to a higher gradient solution only after it completely sunk in the lower gradient solution. The right hemisphere was cryoprotected using gradient sucrose (initially 10% sucrose in 2% PFA, subsequently 20%, 30% sucrose solutions), and the left hemisphere with gradient glycerol and DMSO (10 and 20% glycerol in 2% DMSO). The cryoprotection duration was ~12 days and ~6 days in sucrose and glycerol, respectively. The second goat brain, GB2, was cryoprotected using the 24-h per gradient protocol, where the right hemisphere was cryoprotected using gradient sucrose, and the left hemisphere was protected with gradient glycerol and DMSO.

Both brains, i.e., four frozen blocks, were prepared by the freezing method described above with an isopentane and dry ice bath. To measure the equilibrium temperature (–80°C, the ideal temperature to store the frozen blocks) and freezing rate around the center of the brain samples, we positioned temperature sensors at the centers of goat brain hemispheres (midpoint of the length and width of a hemisphere) at a depth of 15 mm (dorso ventral dimension is ~30 mm) while freezing using the isopentane in a dry ice bath. The freezing rate in goat brain hemispheres that were cryoprotected using gradient sucrose for the 24-h per gradient vs. the equilibrium protocol was measured as 2.2°C/min and 3°C/min, respectively. The brain hemispheres that were cryoprotected using glycerol and DMSO solution for the 24-h per gradient vs. the equilibrium protocol had a freezing rate of 1.7°C/min and 2.3°C/min, respectively. Equilibrium of temperature was achieved after 85 min (GB1–sucrose), 91 min (GB2–sucrose), 90 mins (GB1–glycerol and DMSO), and 83 min (GB2–glycerol and DMSO). The equilibrium temperature in the center of the brains was –78°C.

#### 4.3.1. Histological analysis

The shrinkage in the brain tissue's estimated volume was calculated by measuring the brain dimensions (length × width × height) before and after cryoprotection. A maximum shrinkage of 8% and 2% was measured when the goat brain was cryoprotected using gradient sucrose and glycerol and DMSO combination, respectively.

**Figure 4:**
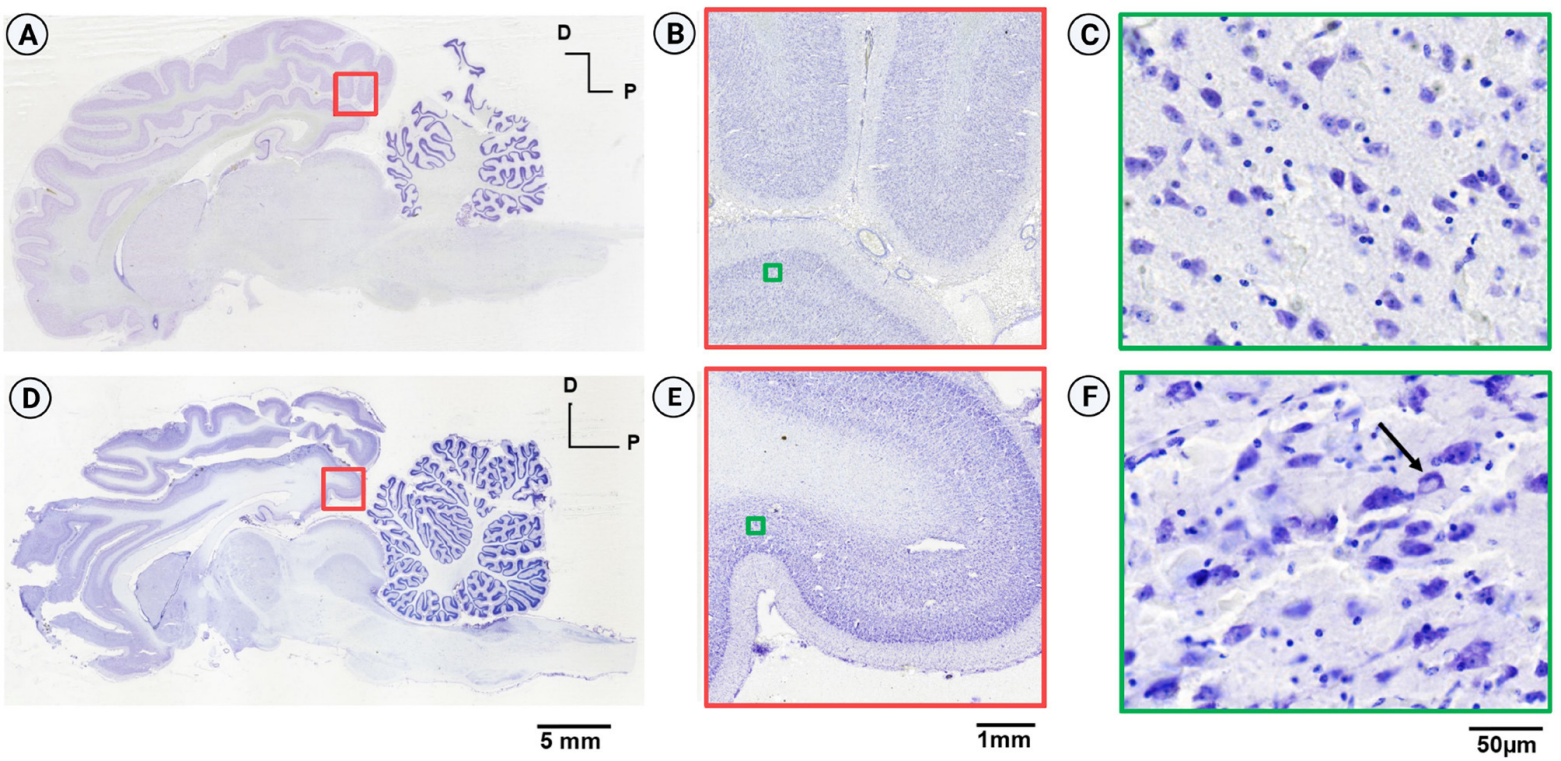
Comparison of Nissl-stained sagittal sections from the goat brain. **(A)** Sagittal section from the right hemisphere cryoprotected with sucrose (equilibrium protocol) displaying good structural integrity and no tissue damage. **(B)** Well-stained section from the occipital cortex. **(C)** Inset from **(B)** exhibits high-resolution imaging, revealing intact neurons without osmotic shock. **(D)** Sagittal section from the left hemisphere, cryoprotected with gradient glycerol (equilibrium protocol), showing poor structural integrity and significant tissue damage. **(E)** Inset from **(D)** depicts a region in the left hemisphere comparable to the right hemisphere, indicating good staining and laminar differentiation. **(F)** High-resolution image demonstrating lack of structural integrity, non-uniform Nissl labeling of neurons, cracks in the extracellular matrix, and the presence of osmotic shock (arrow) due to inadequate cryoprotection.

The tissue quality was assessed for neuronal integrity, signs of fixation damage, osmotic shock, and freezing artifacts. No microbial contamination was observed, indicating good quality of tissue fixation. Figure 4 shows the comparative midsagittal sections of a goat brain (Nissl-stained with Thionin) from both hemispheres. The right hemisphere was cryoprotected in gradient sucrose (Figure 4A), and the left hemisphere in gradient glycerol and DMSO (Figure 4D). The equilibrium method was used in both cases, where the specimens were moved to a higher gradient only after the specimen was completely sunk. Figure 4B shows the uniformly well-stained cerebral cortex section, obtained using sucrose as a cryoprotectant, with no freezing artifacts and intact cortical layers. The high-resolution inset (Figure 4C) shows well-stained and preserved neurons. After using glycerol as a cryoprotectant (Figure 4D), the brain tissue texture was soft and brittle. The high-resolution image in Figure 4F shows poor Nissl labeling, with several cells showing signs of osmotic shock. The overall integrity of the tissue was compromised during sectioning with visible microcracks.

In the second part of the experiment, we compared the duration of cryoprotection for both cryoprotectants using the 24-h protocol, where the specimens were transferred to a higher gradient within 24 h rather than waiting for the tissue to sink completely. The tissue's physical integrity was compromised for both blocks and during cryosectioning, using the tape transfer technique (Pinskiy et al., 2015), significant shattering and tissue loss were observed, indicating the failure of the cryoprotection step due to inadequate time.

### 4.4. Selection of the cryogenic medium or source for freezing

The choice of the cryogenic medium to freeze large brains with frozen block volumes greater than 200 cm^3^ is based on several factors, including spatial uniformity of freezing, the freezing rate, and ease of use. We selected three different modalities for standardizing the freezing of large-volume brain specimens: (a) isopentane in a dry ice bath, (b) liquid nitrogen, and (c) direct freezing in a –80°C freezer (Table 1) as these modalities would provide three different freezing rates within the brain tissue. Liquid nitrogen, due to its extremely low temperature (Umrath, 1974) (–210°C), would provide the fastest cooling rate, followed by isopentane in a dry ice bath (Rosene et al., 1986; Pinskiy et al., 2013, 2015). On the other hand, the -80°C freezer would be the slowest as heat transfers through the air, which is a poor conductor of heat.

**Table 1:** Table includes the list of goat brain specimens processed with varying cryoprotection methods (sucrose, glycerol and DMSO) and freezing media (Isopentane in the dry ice bath, liquid nitrogen, and –80°C freezer).

**Specimen**	**Frozen block volume (cm^3^)**	**Type of cryoprotection (Protocol)**	**Freezing medium**
GB 1 RH	131	Sucrose (Equilibrium)	Isopentane in dry ice
GB 1 LH	142	Glycerol & DMSO (Equilibrium)	Isopentane in dry ice
GB 2 RH	175	Sucrose (24 hrs)	Isopentane in dry ice
GB 2 LH	116	Glycerol & DMSO (24 hrs)	Isopentane in dry ice
GB 3 (Whole brain)	268	Sucrose (Equilibrium)	Isopentane in dry ice
GB 4 (Whole brain)	269	Sucrose (Equilibrium)	Liquid nitrogen
GB 5 (Whole Brain)	269	Sucrose (Equilibrium)	–80°C refrigerator

GB, Goat Brain; RH, Right Hemisphere; LH, Left Hemisphere.

The brain is a heterogeneous tissue composed of different groups of cells such as neurons, glia, white matter tracts, supporting structures such as blood vessels, and water. This heterogeneity results in poor thermal properties for the brain tissue, with a measured thermal conductivity of 0.51 W/mK and a specific heat capacity of 3630 J/Kg.K (Hasgall et al., 2022), making it difficult to freeze the tissue. This problem is exasperated, especially for larger brains. The larger tissue size and its inherent combination of poor thermal properties slow the penetration of the cold front, leading to faster cooling rates at the periphery and slower cooling rates toward the center of the tissue. This non-uniformity in cooling is not much of a concern in smaller brain tissues such as mouse brains, which are effectively frozen using dry ice, isopentane, or liquid nitrogen (Currle and Monuki, 2007; Pinskiy et al., 2013, 2015). Isopentane in a dry ice bath was successfully used to freeze monkey brains with frozen block volumes of up to 150 cm^3^, highlighting the potential for these techniques to be adapted for even larger brain tissues (Rosene et al., 1986; Estrada et al., 2017). The choice of the freezing source should also consider the need to freeze the embedding medium at a similar rate to the brain tissue to prevent the cracking of the tissue.

In addition, finding the optimal freezing rate that minimizes the formation of freezing artifacts in large brain tissues is imperative. The freezing artifacts associated with the preparation of a frozen block, described as having a “Swiss cheese” morphology with holes in the epithelium (interstitial vacuoles along with vacuoles inside the cell cytoplasm) (Chatterjee, 2014; Taqi et al., 2018), are created by the formation of ice crystals that damage cell membranes and compromise the tissue integrity. The freezing rate, particularly in slow freezing, promotes ice crystal formation and expands the solid water content within the tissue specimen, leading to the penetration of membranes (Taqi et al., 2018). The relatively high water fraction in brain tissue (0.75–0.95) means a high degree of ice crystals can potentially form during freezing (Oros-Peusquens et al., 2019). Hence, when placed on a cold surface to freeze without cryoprotection, the brain tissue undergoes a thermal gradient and likely forms crystals, especially in deeper regions, most distant from the freezing surface (Rosene et al., 1986; Watson et al., 1986; Estrada et al., 2017).

**Figure 5:**
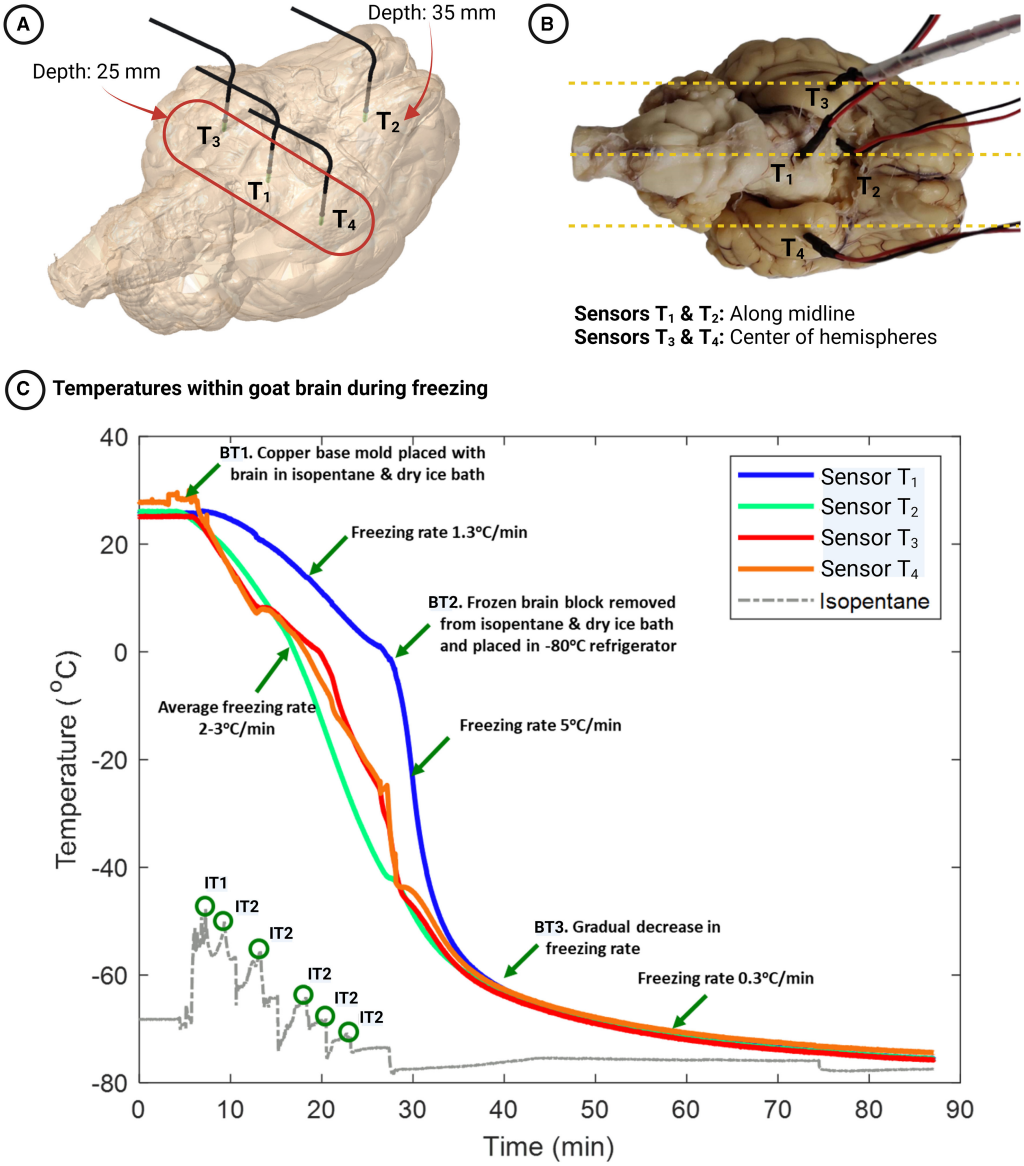
**(A, B)** Locations of temperature sensors within the goat brain - Sensors *T*_1_, *T*_3_, and *T*_4_ are positioned at a depth of 25 mm from the ventral surface, while sensor *T*_2_ is located at a depth of 35 mm. **(C)** Temporal dynamics of temperatures within the goat brain during the freezing process. Event BT1 marks the start of the freezing process (when the brain is kept in an isopentane/dry ice bath). Event BT2 represents the removal of the frozen block from the base mold and placement in a –80°C freezer. Event BT3 indicates a gradual decrease in freezing rate near –62°C. Event IT1 depicts the rise in isopentane temperature when the warmer copper base mold with the brain tissue is placed in the relatively colder isopentane and dry ice bath. Finally, events IT2 correspond to the addition of dry ice to the isopentane bath at regular intervals, as evidenced by the rise and subsequent fall of the isopentane temperature.

We selected gradient sucrose as our method of cryoprotection before freezing and used three whole goat brains (GB3, GB4, GB5) for three choices of the cryogenic medium. The brain tissue, surrounded by an embedding medium, was first placed in the copper base mold. The copper mold was then placed in the freezing source—isopentane/dry ice bath, liquid nitrogen, or –80°C freezer—and the temperature gradients within the brain tissue were monitored using temperature sensors inserted in four different locations. In the example shown in Figures 5A, B, we used an isopentane and dry ice bath as the freezing medium. The temperature sensors *T*_1_, *T*_3_, and *T*_4_ were placed in different places in the goat brain, but all at a depth of 25 mm from the ventral surface of the brain, while sensor *T*_2_ was placed at a depth of 35 mm. Sensors *T*_1_ and *T*_2_ were placed close to the brain's midline to measure the temperatures farthest from the freezing source. Sensors *T*_3_ and *T*_4_ were placed 15 mm on either side of the midline to monitor the temperatures close to the freezing source. The locations were chosen to evaluate the uniformity of freezing within the brain tissue.

### 4.5. Optimal freezing rate with different freezing media

Isopentane cooled to –80°C provides more consistent and reliable results as tested on mouse and monkey brains compared to dry ice alone (Rosene et al., 1986; Kito et al., 2005). In addition, the cooling rate is faster when placing the tissue in a cooling liquid than covering it with pulverized dry ice at the same temperature (Rosene et al., 1986). The faster cooling is because isopentane has a freezing point lower than that of water, allowing it to cool the sample to a lower temperature. Furthermore, its high thermal conductivity enables it to absorb heat more rapidly than dry ice. Hence, the isopentane and dry ice bath acts as an efficient heat sink rather than dry ice alone and cools the tissue rapidly.

Figure 5C shows the temperature gradients within the goat brain while freezing in an isopentane and dry ice bath. Twenty-three minutes after the goat brain was placed in the frozen embedding medium cavity, the temperatures at sensors *T*_1_, *T*_2_, *T*_3_, and *T*_4_ were –1°C, –41°C, –35°C, and –36°C, respectively. The temperature drop was linear for all sensor places, and the freezing rates were 1.3, 3.1, 2.4, and 2.3°C/min for sensors *T*_1_, *T*_2_, *T*_3_, and *T*_4_, respectively (see Figure 5C–Event BT1 to BT2). The frozen block was then removed from the copper mold, covered with dry ice, and placed in a –80°C refrigerator to ensure that the temperatures within the brain would reach this value. Sensor *T*_1_, which was placed close to the midline of the brain and cooled slowly previously, cooled at a faster rate at 5°C/min (see Figure 5C–after Event BT2) until it reached -65°C and then plateaued. Sensor *T*_2_ (placed deeper and close to the midline) and sensors *T*_3_ and *T*_4_ (placed at the center of the right and left hemispheres, respectively) cooled at 2°C/min until the temperatures reached -65°C and plateaued. The cooling rate began to decline at about –63°C as the temperature difference with the source, the isopentane and dry ice bath (~ –76°C), was reduced (see Figure 5C-Event BT3). At all the locations, the cooling rate was 0.3°C/min and stabilized at -76°C after 80 mins of freezing.

Liquid nitrogen is commonly used for freezing biological samples because it can rapidly achieve extremely low temperatures (–210°C) (Umrath, 1974). However, liquid nitrogen, having an extremely low specific heat constant, boils locally at the contact point with any warmer surface, resulting in a vapor barrier that acts as an insulator and slows the penetration of the cold front within the tissue unpredictably (Scouten and Cunningham, 2006). This unequal cooling makes the tissue and the surrounding embedding medium susceptible to cracking. The brain specimen (GB4) frozen using liquid nitrogen developed a severe crack due to rapid freezing at a very low temperature (–210°C), and sectioning and histological evaluation were not possible.

Our third choice was to freeze the sample directly in a –80°C freezer. While this method is simple and requires minimal preparation compared to the other methods, the cooling rate would be the slowest as the freezing source is the air inside the freezer. The freezing rate inside the goat brain (GB5) when the base mold was placed in the –80°C freezer for the entire freezing duration was 0.45°C/min, and all the brain locations stabilized at –75°C in 210 mins. The uniform slow cooling of all block sides caused the embedding medium surfaces to freeze first, including the exposed top surface, resulting in the entrapment of the liquid medium and brain tissue with minimal room for expansion, leading to the cracking of the tissue.

## 5. Discussion

In this paper, we systematically evaluated the choices for the cryoprotectant, the cryoprotection duration, and the cryogenic medium required for preparing large frozen tissue blocks before cryosectioning. Our best results were obtained for specimens that underwent cryoprotection with gradient sucrose using the standard full-time protocol (until it reached equilibrium) and were frozen with isopentane and dry ice bath with the temperature maintained at –78°C to –80°C. The key factors contributing to the successful freezing include the cryoprotectant choice, the cryoprotection duration, the continuous monitoring of the isopentane temperature, the design of a brain master pattern, the use of a sample-specific copper base mold, and the careful choice of the materials used during the freezing process. The optimized protocol was then employed on a large sagittal section (150 × 110 × 42 mm) of the whole human brain, and their cryosections were systematically evaluated. The large-volume brain tissue was sectioned and oriented within the copper base mold, so the maximum depth the cold front had to penetrate was 21 mm, ensuring uniform cooling and resulting in good-quality histology, as described in the results section. The resultant 2100 cryosections were evaluated histologically, and the tissue damage was less than 1% in each section. In addition, the frozen tissue block had a volume of 796 cm^3^, nearly five times larger than the previously frozen large brain blocks (150 cm^3^) (Rosene et al., 1986; Estrada et al., 2017).

For digital histology and 3D reconstruction of large-volume brain specimens, tissues are often processed using thin (5–10 mm) slices, where each slice is frozen in small blocks and cryosectioned (Mancini et al., 2020). In addition, 3D reconstruction of the whole brain volume using digitized tissue sections generated from smaller tissue slices adds more complexity during registration and alignment, as the sheer number of slices can cause misalignment issues (Pichat et al., 2018). Furthermore, a common cutting plane across the different slices needs to be maintained along with minimal tissue loss to reduce the complexities. To address these challenges, our freezing protocol focuses on freezing thick tissue slices (40–50 mm) with minimal tissue damage. This approach of using fewer slices (4–5 slices for an adult human brain) ensures minimal alignment issues during registration and reconstruction, enabling accurate alignment and efficient analysis of the entire brain volume.

### 5.1. Standardization of cryoprotection

The gradient sucrose protocol provided the best results for our tape transfer-based cryosectioning. The gradient glycerol and DMSO cryoprotection was much faster than gradient sucrose and had minimal shrinkage without significant cellular changes, including osmotic shock. This result is consistent with previous reports that 20% gradient glycerol causes minimal shrinkage of the fixed tissue compared to sucrose and replaces water uniformly from the tissue (Rosene et al., 1986; Estrada et al., 2017). However, sectioning of glycerol samples was difficult in the large cryostat at –20°C, specifically using the tape transfer technique (Estrada et al., 2017).

The qualitative analysis of the 24-h cryoprotection protocol showed cellular osmotic shock in several regions, and the frozen block had compromised physical integrity, which was worse for the blocks cryoprotected with glycerol and DMSO. Long-time storage of larger brain specimens in sucrose cryoprotectant is usually not employed due to the likelihood of microbial contaminations (Santos et al., 2014). This is generally solved with sodium azide, often used as a preservative in the buffer solution, which aids cellular protection and microbial contamination (Minassian and Huang, 1979). We did not use sodium azide for our specimens but stored them at 4°C with stable pH and microbial growth monitoring.

### 5.2. Cryogenic source and freezing rate

The temperature distribution and varying freezing rates in different locations within the goat brain helped us understand the thermodynamic process parameters, namely the freezing source and the freezing rate, for freezing large brains. The freezing rates within the goat brain using the isopentane and dry ice bath were, on average, 2.7°C/min over 30 mins (based on a regression analysis over the period where the temperature drop is nearly linear). Although the freezing rate at sensor *T*_1_ was initially lower at 1.3°C/min, the rate increased on placing the frozen block inside the –80°C refrigerator, resulting in an average freezing rate of 2.8°C/min. The temperatures at the four locations within the goat brain reached –60°C simultaneously (30 mins), ensuring uniformity in freezing rates within the brain tissue. When frozen using an isopentane bath, a freezing rate of 3.5°C/min inside an albumin-gelatin block (81 cm^3^) was reported (Rosene et al., 1986). Although the freezing rate in our case was slightly lower than that reported, the volume of the frozen block was much higher (~270 cm^3^). As the lateral surface of the brain was placed over the frozen embedding medium, the maximum depth the cold front had to penetrate was approximately 25 mm, taking into account half the goat brain width. Therefore, an average cooling rate of about 2–3°C/min in the brain tissue is required to ensure minimal freezing artifacts are present. Also, uniformity of freezing rate throughout the tissue ensures an intact frozen brain devoid of cracks.

The cold front within the tissue propagates from five directions (all faces of the base mold except the top, which is exposed to ambient air of –21°C within the styrofoam box) when frozen in isopentane and dry ice bath. Therefore, cooling the surface exposed to the warmer environment is slow and allows for the expansion of the tissue embedding medium during freezing. The cooling rate improved considerably once the block was placed in a –80°C freezer, as the cold front can propagate from all six directions of the frozen block. The equilibrium points within the goat brain (–76°C) were achieved 80 mins after the start of freezing—23 mins for embedding medium freezing and 57 mins inside the –80°C freezer. Thus, the optimal procedure for freezing large brain tissue should ensure quick cooling of the embedding medium around the brain tissue, immediate removal of the frozen block from the copper base mold, and transfer to the –80°C refrigerator, ensuring faster cooling within the brain.

When the goat brain was directly frozen in the –80°C freezer, the top surface of the embedding medium froze along with the rest of the surfaces in contact with the copper base mold. With the top surface frozen, a considerable amount of embedding medium is trapped inside as a liquid, confirmed by the little caving-in reaction of the frozen surface when pressed by hand. Once all the embedding medium froze with minimal space for expansion, it damaged the brain tissue, and cracks developed. Therefore, freezing in an isopentane and dry ice bath produced blocks ideal for cryosectioning without any artifacts.

### 5.3. Freezing protocol: component design and material selection

The material and manufacturing processes selected for the brain master pattern and the base mold play a vital role during the freezing process. 3D models are generally generated from Magnetic Resonance Imaging (MRI) models (Pinskiy et al., 2015; Guy et al., 2016; Lin et al., 2019) (with millimeter accuracy) while scanning of the brain surface (40-micron accuracy) produces superior 3D models (see Figure 1B1). Our brain master pattern was manufactured using metal 3D printing (see Figure 1B2) to capture finer details and accurately represent its surface. An aluminum alloy with a high thermal conductivity of 160 W/mK (Sélo et al., 2020) was selected for manufacturing the master pattern as it can be quickly heated to be extracted from the frozen embedding medium.

The master pattern creates a negative cavity in a frozen embedding medium, thus helping the alignment of the brain tissue in neuroanatomical coordinates appropriately (see Figure 1C2). Neuroanatomical coordinates provide a standard reference system for locating specific brain structures across individuals and species. Proper alignment of the brain in neuroanatomical coordinates within the embedding medium before freezing is critical to avoid issues during cryosectioning and 3D reconstruction studies. It involves positioning the tissue in a specific orientation, such as dorsal-ventral or rostral-caudal, and ensuring it is level and centered in the freezing container. The brain tissue lacks rigid edges that can help align it with a fixed reference (Pinskiy et al., 2013). In addition, the opacity of the frozen embedding medium makes it difficult to infer the brain tissue's orientation within the block (Pinskiy et al., 2013). Even a slight deviation in alignment can lead to uneven sectioning and complicate the identification of specific brain regions of large brain tissues. The brain master pattern and the copper base mold provide a simple and reproducible way of placing the brain in a shape-specific cavity relative to the edges of the base mold. The rectangular edge of the frozen block is used as a reference during cryostat sectioning.

The base mold (see Figure 1B3) was manufactured using copper (thermal conductivity of 386 W/mK) to provide fast freezing rates. More importantly, the tissue-freezing medium has thermal properties comparable to brain tissue and is a poor thermal conductor. It is, therefore, essential to minimize the embedding medium surrounding the tissue, as the rate of thermal heat transfer by conduction is inversely proportional to the thickness of the medium. However, the rigidity of the compound and support for the tissue need to be ensured. Given these constraints, the internal dimensions of the copper base mold were designed to be 5–10% more than the dimensions of the brain. When the copper base mold was placed in the isopentane/dry ice bath, there was a rapid increase in isopentane temperature from –75°C to –65°C due to the heat transfer from the warmer base mold (see Figure 5C–Event IT1). Thus, monitoring the isopentane temperature continuously and adding dry ice at regular intervals helps maintain the isopentane temperature close to –75°C (see Figure 5C–Event IT2) and the freezing rate.

By incorporating these engineering advancements, we provide a valuable resource method to bridge the gap between traditional neuroscience approaches and the challenges of studying the structure and connectivity of large brains at a cellular level. The majority of human connectomics studies have predominantly relied on computational neurobiology and magnetic resonance imaging approaches. However, our method of freezing large-volume brains, utilizing engineering technologies, presents a standardized and scalable approach for conducting single-cell level connectomics studies with minimal errors. Our interdisciplinary approach combining fundamental aspects of neuroscience and engineering has the potential to significantly advance our understanding of brain connectivity and pave the way for new discoveries in neuroscience.

## Data availability statement

The original contributions presented in the study are included in the article/supplementary material, further inquiries can be directed to the corresponding author.

## Ethics statement

The animal study and the study involving human brain samples were approved by the Institutional Ethics Committee, Indian Institute of Technology Madras, India (IEC/2021-01/MS/06), and Office of Research—Institutional Review Board, Christian Medical College, Vellore, India (IRB Min. No. 13262). The studies were conducted in accordance with the local legislation and institutional requirements. Written informed consent was obtained from the next of kin before extracting the human brain sample used in this study.

## Author contributions

RK: Conceptualization, Formal analysis, Investigation, Methodology, Writing—original draft, Visualization, Writing—review & editing. RV: Conceptualization, Formal analysis, Investigation, Methodology, Writing—original draft, Project administration, Writing—review & editing. KP: Investigation, Validation, Visualization, Writing—review & editing. JR: Investigation, Validation, Visualization, Writing—review & editing. SP: Investigation, Resources, Validation, Writing—review & editing. SS: Resources, Writing—review & editing, Investigation. JJa: Investigation, Resources, Validation, Writing—review & editing. MB: Resources, Writing—review & editing, Investigation. PM: Resources, Supervision, Writing—review & editing, Conceptualization, Funding acquisition. JJo: Funding acquisition, Project administration, Resources, Supervision, Writing—review & editing, Conceptualization, Methodology. MS: Funding acquisition, Project administration, Resources, Supervision, Writing—review & editing, Conceptualization.
